# Intensive lifestyle intervention in type 2 diabetes and risk of incident coronary artery disease for the common haptoglobin phenotypes: the Look AHEAD study

**DOI:** 10.1186/s12933-024-02164-8

**Published:** 2024-02-24

**Authors:** Rachel A. Warren, Michael P. Bancks, Allie S. Carew, Andrew P. Levy, John Sapp, Judy Bahnson, Cora E. Lewis, Eric B. Rimm, Mark A. Espeland, Leah E. Cahill

**Affiliations:** 1 Department of Medicine, Dalhousie University, Halifax, Canada; 2 QEII Health Sciences Centre, Nova Scotia Health Authority, Halifax, Canada; 3 Department of Epidemiology and Prevention, Wake Forest University School of Medicine, Winston-Salem, USA; 4 Department of Community Health and Epidemiology, Dalhousie University, Halifax, Canada; 5 Rappaport Faculty of Medicine, Technion Israel Institute of Technology, Haifa, Israel; 6 Department of Biostatistics and Data Science, Wake Forest University School of Medicine, Winston-Salem, USA; 7 Department of Epidemiology, University of Alabama at Birmingham, Birmingham, USA; 8 Department of Nutrition, Harvard T. H. Chan School of Public Health, Boston, USA; 9 Department of Epidemiology, Harvard T. H. Chan School of Public Health, Boston, USA; 10 Department of Internal Medicine-Gerontology and Geriatric Medicine, Wake Forest University School of Medicine, Winston-Salem, USA

**Keywords:** Coronary artery disease, Epidemiology, Genetic association, Glycated hemoglobin, Haptoglobin phenotype, Type 2 diabetes mellitus

## Abstract

**Background:**

Intensive glycemic control reduced coronary artery disease (CAD) events among the Action to Control Cardiovascular Disease Risk in Diabetes (ACCORD) participants with the haptoglobin (Hp) 2-2 phenotype only. It remains unknown whether Hp phenotype modifies the effect of an intensive lifestyle intervention (ILI) on CAD in type 2 diabetes.

**Methods:**

Haptoglobin phenotype was measured in 4542 samples from the Action for Health in Diabetes (Look AHEAD) study. Cox regression models assessed the effect of ILI (focused on weight loss from caloric restriction and physical activity) versus diabetes support and education (DSE) on CAD events in each phenotype group, and within pre-specified subgroups including race/ethnicity, sex, history of cardiovascular disease, diabetes medication use, and diabetes duration.

**Results:**

1590 (35%) participants had the Hp2-2 phenotype. The ILI did not lower glycated hemoglobin (%HbA1c) to < 6.5% in either phenotype, with a peak significant difference between treatment arms of 0.5% [non-Hp2-2] and 0.6% [Hp2-2]. The cumulative CAD incidence was 13.4% and 13.8% in the DSE arm and 12.2% and 13.6% in the ILI arm for non-Hp2-2 and Hp2-2 groups, respectively. Compared to DSE, the ILI was not associated with CAD among participants without (HR = 0.95, 95% CI 0.78–1.17) or with (0.89, 0.68–1.19) the Hp2-2 phenotype (p-interaction between Hp phenotype and ILI = 0.58). After Bonferroni correction, there were no significant results among any subgroups.

**Conclusions:**

Hp phenotype did not modify the effect of the weight loss ILI on risk of CAD in Look AHEAD, potentially because it did not substantially impact glycemic control among participants with or without the Hp2-2 phenotype. Further research is needed to determine if these results are conclusive.

**Supplementary Information:**

The online version contains supplementary material available at 10.1186/s12933-024-02164-8.

## Background

People with type 2 diabetes have an increased risk of cardiovascular disease (CVD) morbidity and mortality when compared to people without diabetes [1]. Weight loss is recommended for individuals with type 2 diabetes due to its ability to improve multiple clinical risk factors for cardiovascular morbidity and mortality, including glycemic control [2, 3]. The Action for Health in Diabetes (Look AHEAD) study was designed to determine whether randomization to an intensive lifestyle intervention (ILI) aiming to achieve weight loss through caloric restriction and increased physical activity decreased cardiovascular disease morbidity and mortality when compared to diabetes support and education (DSE) among adults with type 2 diabetes who had overweight/obesity [4, 5]. Although there were differences in cardiovascular disease risk factors (including glycated hemoglobin (HbA1c)) between randomization arms during the study, the ILI did not reduce the overall risk of CVD when compared to DSE [4]. A potential explanation is that the ILI may only be effective in reducing CVD risk in a subset of people with type 2 diabetes. Unmeasured differences between participants, such as genetics, that affect the relationship between modifiable risk factors and CVD could help to explain the results of the Look AHEAD trial.

A common variation in the gene that codes for the abundant plasma protein haptoglobin (Hp) identifies individuals who may be at increased risk of coronary artery disease (CAD, such as myocardial infarction) from hyperglycemia [6–9]. In hyperglycemia, the antioxidant capabilities of the Hp protein are impaired among people with the Hp2-2 phenotype (~ 40% worldwide [10]) relative to the non-Hp2-2 phenotypes (Hp1-1and Hp2-1), and high-density lipoprotein (HDL) has been shown to be dysfunctional and pro-atherogenic with the potential to increase susceptibility to atherosclerosis, and ultimately CAD [7, 11–13]. As a result, glycemic control may be particularly important for CAD prevention among people with the Hp2-2 phenotype and hyperglycemia, among whom Hp function is impaired. The Hp phenotype frequencies differ by geographic location and race/ethnicity and may potentially explain the results of previous clinical trials that have not demonstrated CAD benefit from intensive glycemic control [4, 14, 15].

We recently found that intensive glycemic control (targeting HbA_1c_ < 6.0%) was effective at preventing incident CAD events when compared to standard therapy (targeting HbA_1c_ of 7.0–7.9%) among White participants with the Hp2-2 phenotype in the Action to Control Cardiovascular Risk in Diabetes (ACCORD) study [16]. No benefit was observed among ACCORD participants without the Hp2-2 phenotype who had increased mortality risk from intensive therapy [16]. These findings provide evidence to support using Hp phenotype as a biomarker to help determine the use of therapies (such as intensive glycemic control) that could help reduce CAD in patients with type 2 diabetes. However, the ACCORD study used pharmacotherapy for intensive glucose lowering and it remains unknown whether the effects of a lifestyle intervention on CAD risk in type 2 diabetes would similarly be influenced by Hp phenotype.

The primary objective of the present study was to determine whether the effect of an intensive lifestyle intervention for weight loss (reduced caloric intake and increased physical activity) versus diabetes support and education on CAD risk is dependent on haptoglobin phenotype in the Look AHEAD study. We also assessed for heterogeneity of this association within pre-specified demographic and clinical subgroups including race/ethnicity, sex, history of cardiovascular disease, diabetes medication use, and diabetes duration.

## Methods

### Study design and participants

A re-analysis of data from the Look AHEAD study with the addition of Hp phenotype measurement was undertaken to determine the relationship between the weight loss ILI (compared to DSE) and CAD events among each of the Hp phenotype groups separately. The design, methods and major findings of the Look AHEAD study (ClinicalTrials.gov identifier: NCT00017953) have been reported previously [4, 5]. Briefly, 5145 patients with type 2 diabetes were recruited between August 2001 and April 2004. Participants were aged 45–75 and had a body-mass index (BMI) of 25 kg/m^2^ or more (27 or more in participants taking insulin), a glycated hemoglobin (HbA_1c_) of 11% or less, a systolic blood pressure of < 160 mmHg, a diastolic blood pressure < 100 mmHg, a triglyceride level of less than 600 mg/dL, the ability to complete a valid maximal exercise test and an established relationship with a primary care provider. Participants with and without a history of cardiovascular disease were included [4, 5]. Participants were randomized to receive either ILI (aimed at achieving and maintaining weight loss of at least 7% by focusing on reduced caloric intake and increased physical activity) or to receive DSE over a median follow-up of 9.6 years. The ILI included group and individual counseling sessions, which occurred weekly during the first 6 months with decreasing frequency over the course of the trial. Specific intervention strategies included a calorie goal of 1200 to 1800 kcal per day (with < 30% of calories from fat and > 15% from protein), the use of meal-replacement products, and at least 175 min of moderate-intensity physical activity per week. A toolbox of strategies was available for participants having difficulty achieving the weight-loss goals. DSE included three group sessions per year focused on diet, exercise, and social support during years 1 through 4, and annually thereafter. Each participating center obtained ethical approval, and all participants provided written informed consent [4, 5].

### Haptoglobin phenotyping

Hp phenotyping was performed using a validated high throughput enzyme linked immunosorbent assay (ELISA) that can distinguish the Hp2-2 protein from the non-Hp2-2 proteins with a sensitivity and specificity of 99% and 98.1% respectively [17]. The ELISA identifies Hp phenotypes based on the differences in Hp protein size/structure [17]. There is a 1:1 correspondence between Hp genotype and Hp phenotype [18]. Hp phenotype does not change over time; therefore, a blood sample from any follow-up visit was used. Of the 5145 Look AHEAD participants, a serum sample was available for Hp phenotyping for 4542 (88.3%). The remaining 603 participants were excluded because serum samples from these participants were not available due to the depletion of samples from other studies or due to consent limitations.

### Outcome

Our primary outcome of major CAD events was defined as a composite of the following pre-specified Look AHEAD outcomes [4, 5]: fatal and non-fatal MI, hospitalization for angina, and fatal CAD (definite and probable). An independent adjudication committee validated all outcome events [4]. Although the mechanism is not well understood, stroke is an endpoint that has been associated with the Hp1-1 phenotype rather than the Hp2-2 phenotype [19, 20]. Stroke is a composite of different stroke subtypes with different etiologies that are not always related to atherosclerosis, suggesting that CAD and stroke should be separated from a composite CVD outcome for analyses by Hp phenotype. Therefore, the present analysis studied the primary outcome of CAD events rather than the original Look AHEAD study primary outcome of a composite of death from cardiovascular causes, nonfatal myocardial infarction, non-fatal stroke, or hospitalization for angina.

In a sensitivity analysis, we also investigated the relationship between the ILI and other Look AHEAD outcomes including the study primary composite outcome of CVD (death from cardiovascular causes, nonfatal myocardial infarction, nonfatal stroke, or hospitalization for angina), total mortality, and severe hypoglycemia events (loss of consciousness, seizure, or a glucose < 70 mg/dL that prevented self-treatment and required assistance of another person) (Additional file 1: Table S1).

### Statistical analysis

All analyses were conducted using STATA/SE software version 18 (StataCorp, College Station, TX). With the exception of when testing for Hardy Weinberg equilibrium (HWE), the common approach of dichotomizing the Hp2-2 phenotype variable to represent Hp2-2 phenotype (yes/no) was used because of the low frequency of the Hp1-1 phenotype and the similar structure and function of Hp1-1 and Hp2-1 relative to Hp2-2 [8, 9, 16, 21, 22].

Participants were grouped based on a combination of their treatment assignment and Hp phenotype, and baseline characteristics were summarized using t tests, or Kruskal-Wallis tests for continuous variables and x^2^ tests for categorical variables.

The goal of the present analysis was to replicate the original Look AHEAD study [4] analysis as closely as possible within each phenotype group. As such, cause-specific Cox proportional hazards regression models were used to assess the effect of the intervention on incident CAD, as is recommended for etiological regression analyses even with competing risks [23, 24]. We assessed for effect modification of this association by haptoglobin phenotype by including an interaction term between intervention group and Hp phenotype and stratifying our results by Hp phenotype. Multivariable models were adjusted for traditional risk factors as well as any other variables that were different between treatment groups at baseline. The frequency of the Hp2-2 phenotype differs among race-based and geographic populations [10] and so race/ethnicity was also identified as an important variable to be included in the model. As such, models were adjusted for age, sex, study site, previous CVD, race/ethnicity (for model in all participants), triglycerides, systolic blood pressure, diastolic blood pressure, income (with category for missing), education, diabetes medication use, anti-hypertensive medication use, lipid medication use, and antidepressant medication use.

We identified a priori sub-groups for stratification of our primary analyses for this study. Current reporting guidelines recommend disaggregation of results by sex [25], and the distribution of the Hp phenotype frequencies differ among race-based and geographic populations [10]. Current diabetes care guidelines suggest that diabetes duration and established CVD are important factors in glucose management [26], Thus, stratified analyses by race/ethnicity, sex, previous CVD at baseline, diabetes duration (> 10 years), and diabetes medication use at baseline were performed in each phenotype group separately. For the race/ethnicity stratification, we were only able to run the adjusted model in the following race/ethnic groups with sufficient numbers for valid estimates and to ensure anonymity: White (67% of total sample), Black (17%) and Hispanic (13%). Interactions were tested between ILI and race/ethnicity, sex, CVD history at baseline, diabetes duration, and diabetes medication use by adding an interaction term to the model for each phenotype group.

On September 14, 2012, on the basis of a futility analysis and recommendation from the data and safety monitoring board, the Look AHEAD intervention was stopped and all data were censored at that date [4]. Follow-up time for the current analysis was defined as the time from date of randomization to date of documented outcome, or until a participant was censored if no event occurred. To account for multiple testing, we applied a Bonferroni corrected significance level of *P* < 0.002 (0.05 divided by 24). In a sensitivity analysis, inverse probability weighting was used to assess the impact for potential selection bias of excluding individuals missing Hp phenotype data from all enrolled participants (11.7%) [27]. In another sensitivity analysis, we restricted follow-up to years 1, 3 and 5.

## Results

**Fig. 1:**
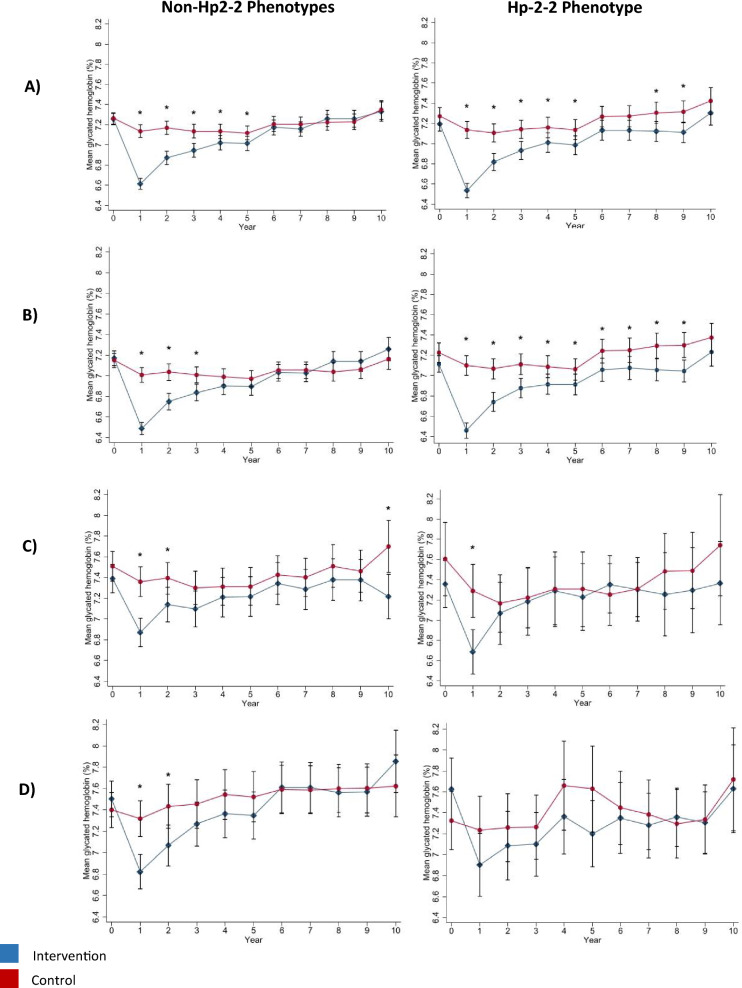
Mean glycated hemoglobin over study duration by treatment group overall and in each of the three largest race groups (White, Black, Hispanic), in each Hp phenotype group separately. Mean glycated hemoglobin (%) levels by treatment group over study duration among (**A**) all participants, (**B**) White participants, (**C**) Black participants, and (**D**) Hispanic participants. Mean glycated hemoglobin at each time-point was compared between treatment groups using t-tests, asterisks indicate P < 0.05 for the between-group comparison

**Fig. 2:**
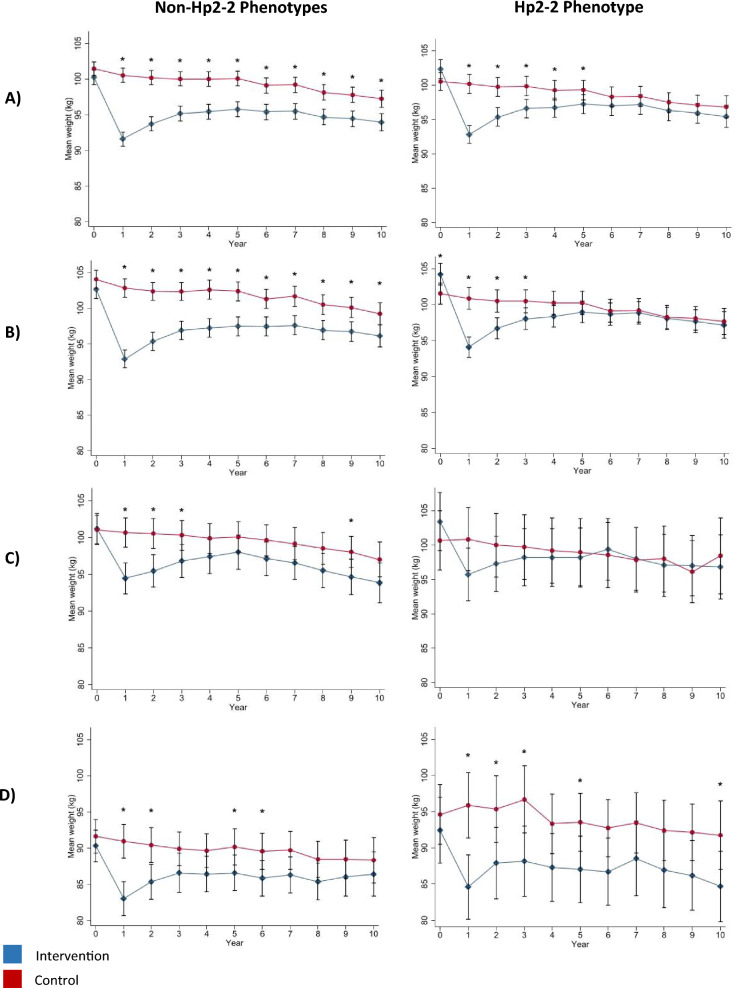
Mean weight over study duration by treatment group overall and in each of the three largest race groups (White Black, Hispanic), in each Hp phenotype group separately. Mean weight (kg) by treatment group over study duration among (**A**) all participants, (**B**) White participants, (**C**) Black participants, and (**D**) Hispanic participants. Mean weight at each time-point was compared between treatment groups using t-tests, asterisks indicate P < 0.05 for the between-group comparison

The frequencies of the Hp phenotypes were 19.7% Hp1-1 (n = 897), 45.2% Hp2-1 (n = 2055), 35% Hp2-2 (n = 1590), and were not in HWE (p-value < 0.01). The mean follow-up was 9.7 years for each of the Hp phenotype groups. Baseline characteristics that differed either between treatment groups or between phenotype groups included: sex, race/ethnicity, education, history of CVD, beta-blocker use, diuretic use, any anti-hypertensive medication use, any lipid-lowering medication use, anti-depressant medication use, systolic and diastolic blood pressure, triglycerides, and income (Table 1). 9.8% of data were missing for income and a category for missing was used for the income variable. Less than 4% of data were missing for any other baseline variables. Mean HbA_1c_ and weight over study duration by intervention group for each phenotype group are shown in Figs. 1 and 2 respectively. Overall, the peak difference in mean HbA_1c_ comparing ILI to DSE for each of the non-Hp2-2 and Hp2-2 groups was 0.5 and 0.6% respectively at year 1, a non-substantial difference that slowly diminished over the course of the study (Fig. 1). The peak difference in mean weight for the ILI compared to DSE was 8.9 and 7.4 kg (8.9 and 7.4 percent) for each of the non-Hp2-2 and Hp2-2 groups respectively at year 1. A significant difference in mean weight between study groups was maintained for the duration of the study for the non-Hp2-2 phenotype group only (Fig. 2).

**Table 1:** Baseline characteristics^a^ stratified by diabetes support and education (DSE) and intensive lifestyle intervention (ILI) treatment group and Hp phenotype in the Look AHEAD Study

	Non-Hp2-2 phenotypes				Hp2-2 phenotype				
	All (n = 2952)	DSE (n = 1478)	ILI (n = 1474)	P-value	All (n = 1590)	DSE (n = 767)	ILI (n = 823)	P-value	Overall P-value^**^
Characteristic									
Age, years	58.9 ± 6.8	59.2 ± 6.8	58.7 ± 6.7	0.09	58.8 ± 6.7	58.8 ± 6.7	58.8 ± 6.7	0.91	0.51
Female sex, *n* (%)	1773 (60.1)	878 (59.4)	895 (60.7)	0.47	906 (57.0)	448 (58.4)	458 (55.7)	0.27	0.04
Race, *n* (%)				0.96				0.63	< 0.01
Black	583 (19.8)	301 (20.4)	282 (19.1)		167 (10.5)	73 (9.5)	94 (11.4)		
Native American	13 (0.4)	6 (0.4)	7 (0.5)		10 (0.6)	4 (0.5)	6 (0.7)		
Asian or Pacific islander	22 (0.8)	10 (0.7)	12 (0.8)		23 (1.5)	9 (1.2)	14 (1.7)		
White	1833 (62.1)	914 (61.8)	919 (62.4)		1197 (75.3)	584 (76.1)	613 (74.5)		
Hispanic	431 (14.6)	214 (14.5)	217 (14.7)		165 (10.4)	81 (10.6)	84 (10.2)		
Unspecified	69 (2.3)	33 (2.2)	36 (2.4)		28 (1.8)	16 (2.1)	12 (1.5)		
Education, *n* (%)				0.31				0.71	0.02
High-school or less	579 (19.6)	283 (19.2)	296 (20.1)		263 (16.5)	125 (16.3)	138 (16.8)		
Some college	856 (29.0)	434 (29.4)	442 (28.7)		443 (27.9)	212 (27.6)	231 (28.1)		
College graduate	889 (30.1)	451 (30.5)	438 (29.8)		511 (32.1)	242 (31.6)	269 (32.7)		
Graduate school	559 (19.0)	269 (18.2)	290 (19.7)		343 (21.6)	170 (22.2)	173 (21.0)		
Other	67 (2.3)	41 (2.8)	26 (1.8)		30 (1.9)	18 (2.4)	12 (1.5)		
History of CVD, *n* (%)	408 (13.8)	205 (13.9)	203 (13.8)	0.94	213 (13.4)	89 (11.6)	124 (15.1)	0.04	0.69
Current smoking, *n* (%)	117 (4.0)	54 (3.7)	63 (4.3)	0.39	62 (3.9)	29 (3.8)	33 (4.0)	0.82	0.91
Income in last year, *n* (%)				0.83				0.37	< 0.01
< $20,000	318 (10.8)	155 (10.5)	163 (11.1)		126 (7.9)	64 (8.3)	62 (7.5)		
$20,000–$39,999	570 (19.3)	294 (19.9)	276 (18.7)		289 (18.2)	130 (17.0)	159 (19.3)		
$40,000–59,999	554 (18.8)	271 (18.3)	283 (19.2)		296 (18.6)	144 (18.8)	152 (18.5)		
$60,000–$79,999	453 (15.4)	219 (14.8)	234 (15.9)		231 (14.5)	115 (15.0)	116 (14.1)		
≥ $80,000	765 (25.9)	387 (26.2)	378 (25.6)		497 (31.3)	251 (32.7)	246 (29.9)		
Missing	292 (9.9)	152 (10.3)	140 (9.5)		151 (9.5)	63 (8.2)	88 (10.7)		
Medications, *n* (%)									
Insulin	442 (15.5)	230 (16.1)	212 (14.9)	0.38	239 (15.57)	110 (15.0)	129 (16.1)	0.58	0.94
Metformin	1548 (53.6)	755 (52.2)	793 (55.0)	0.13	867 (55.5)	427 (57.1)	440 (54.0)	0.21	0.22
Sulfonylurea	1342 (46.7)	681 (47.1)	661 (46.2)	0.63	702 (45.4)	326 (44.2)	376 (46.4)	0.38	0.41
Thiazolidinedione	795 (27.7)	405 (28.1)	390 (27.2)	0.62	416 (27.1)	212 (29.0)	204 (25.4)	0.11	0.71
Any diabetes medication	2544 (87.0)	1269 (86.6)	1275 (87.3)	0.57	1357 (86.1)	651 (86.0)	706 (86.2)	0.91	0.41
Beta-blocker	640 (21.7)	316 (21.4)	324 (22.0)	0.69	369 (23.2)	161(21.0)	208 (25.3)	0.04	0.24
ACE inhibitor	1261 (43.7)	647 (44.7)	614 (42.6)	0.26	694 (45.0)	331 (45.2)	363 (44.8)	0.87	0.40
Angiotensin receptor blocker	465 (16.3)	224 (15.6)	241 (16.9)	0.35	262 (17.1)	122 (16.7)	140 (17.4)	0.74	0.49
Diuretic	949 (33.0)	496 (34.4)	453 (31.7)	0.12	471 (30.5)	197 (26.9)	274 (33.8)	< 0.01	0.09
Any anti-hypertensive medication	2127 (72.9)	1066 (73.0)	1061 (72.8)	0.93	1148 (73.6)	540 (72.8)	608 (74.3)	0.49	0.62
Statins	1285 (44.5)	632 (43.5)	653 (45.5)	0.27	763 (49.1)	363 (49.1)	400 (49.1)	0.99	< 0.01
Any lipid-lowing medication	1426 (49.3)	701 (48.2)	725 (50.5)	0.22	841 (54.1)	404 (54.6)	437 (53.6)	0.7	< 0.01
Anti-depressant	476 (16.6)	205 (14.3)	271 (19.0)	< 0.01	286 (18.6)	136 (18.6)	150 (18.6)	0.99	0.10
Weight, kg	100.9 ± 19.3	101.5 ± 19.2	100.3 ± 19.5	0.09	101.5 ± 19.2	100.5 ± 18.4	102.3 ± 19.8	0.06	0.32
BMI, kg/m^2^	36.0 ± 5.9	36.1 ± 5.8	35.9 ± 6.0	0.21	35.9 ± 5.8	35.8 ± 5.7	36.0 ± 6.0	0.42	0.57
Waist circumference, cm	113.8 ± 14.2	114.2 ± 14.0	113.5 ± 14.4	0.21	114.0 ± 13.7	113.6 ± 12.9	114.3 ± 14.5	0.33	0.75
Glycated hemoglobin									
Mean	7.3 ± 1.1	7.3 ± 1.1	7.3 ± 1.1	0.90	7.2 ± 1.2	7.3 ± 1.2	7.2 ± 1.1	0.2	0.43
Median (IQR)	7.0 (6.5–7.8)	7.1 (6.5–7.8)	7.0 (6.4–7.9)		7.0 (6.4–7.8)	7.0 (6.4–7.8)	7.0 (6.4–7.8)		
Blood pressure, mmHg									
Systolic	129.4 ± 17.3	130.1 ± 17.2	128.6 ± 17.3	0.01	128.8 ± 17.0	129.1 ± 16.7	128.5 ± 17.3	0.43	0.27
Diastolic	70.2 ± 9.7	70.5 ± 9.7	69.8 ± 9.6	0.04	70.3 ± 9.5	70.4 ± 9.6	70.3 ± 9.3	0.91	0.59
HDL-cholesterol, mg/dL	42.7 ± 11.9	43.5 ± 11.7	43.9 ± 12.2	0.32	43.3 ± 11.7	43.8 ± 12.1	42.8 ± 11.4	0.08	0.26
LDL-cholesterol, mg/dL	112.7 ± 32.6	112.0 ± 32.4	113.3 ± 32.8	0.28	111.7 ± 31.3	112.9 ± 31.6	110.6 ± 31.0	0.14	0.33
Triglycerides, mg/dL				0.15				0.18	0.04
Median (IQR)	152 (105–219)	152 (105–217)	151 (106–120)		157 (111–223)	151 (109–221)	162 (113–225)		

*ACE* angiotensin-converting enzyme inhibitor, *CVD* cardiovascular disease, *HDL* high-density lipoprotein, *Hp* haptoglobin, *IQR* interquartile range, *LDL* low-density lipoprotein

^a^Plus-minus values are means ± SD

^**^P-value comparing characteristics between Hp phenotypes

The 10 year cumulative incidence of CAD in the DSE arm was 13.4% for those without Hp2-2 and 13.8% for those with Hp2-2, and in the ILI arm was 12.2% for those without Hp2-2 and 13.6% for those with Hp2-2. When compared to DSE, ILI was not associated with CAD risk among participants with the non-Hp2-2 phenotype (hazard ratio [HR] = 0.95, 95% CI 0.78–1.17) or the Hp2-2 phenotype (0.89, 0.68–1.19, p-interaction = 0.58) (Table 2). No significant results were observed for any subgroup in either phenotype group after Bonferroni correction for multiple comparisons (Table 3). A sensitivity analysis using inverse probability weighting to account for missing Hp phenotype data yielded comparable results (data not shown).

**Table 2:** Multivariable adjusted hazard ratios (aHR) for CAD^a^ events comparing assignment to intensive lifestyle intervention versus diabetes support and education control groups for each phenotype group separately

	DSE (reference)		ILI		Hazard ratios (HRs)			
	# Events/n	Person-years	# Events/n	Person-years	uHR (95% CI)	P-value	aHR^b^ (95% CI)	P-value
Non-Hp2-2 phenotypes	198/1478	13,338.33	180/1474	13,433.02	0.90 (0.74–1.10)	0.32	0.95 (0.78–1.17)	0.66
Hp2-2 phenotype	106/767	6940.46	112/823	7391.85	0.99 (0.76–1.30)	0.96	0.89 (0.68–1.19)	0.44

*CAD* coronary artery disease, *CI* confidence interval, *DSE* diabetes support and education, *Hp* haptoglobin, *aHR* adjusted hazard ratio, *uHR* unadjusted hazard ratio, *ILI* intensive lifestyle intervention

^a^The CAD event outcome is a composite of fatal and non-fatal MI, hospitalization for angina, and possible fatal CAD

^b^Models were adjusted for age, sex, race, study site, prior history of CVD, triglycerides, systolic blood pressure, diastolic blood pressure, income (with category for missing), education, antidepressant medication use, any diabetes medication use, any anti-hypertensive medication use and any lipid medication use. After Bonferroni correction to account for multiple testing, a *P*-value threshold of < 0.002 (0.05 divided by 24) was used

The P-value for the test of interaction between intervention and Hp phenotype for the adjusted model is 0.58

**Table 3:** Stratified multivariable-adjusted hazard ratios for risk of CAD^a^comparing intensive lifestyle intervention to diabetes support and education control group by haptoglobin phenotype group

	DSE (Reference)		ILI		Hazard Ratios (HRs)				
	# Events/n	Person-years	# Events/n	Person-years	uHR (95% CI)	P-value	aHR^b^ (95% CI)	P-value	P-interaction^†^
**Non-Hp2-2 Phenotype**									
By race									0.32
White (n = 1833)	146/914	8138.26	129/919	8321.71	0.86 (0.68–1.09)	0.22	0.89 (0.69–1.13)	0.33	
Black (n = 583)	23/301	2799.44	35/282	2564.85	1.67 (0.98–2.82)	0.06	2.16 (1.20–3.90)	0.01	
Hispanic (n = 431)	23/214	1961.51	11/217	2052.12	0.45 (0.22–0.93)	0.03	0.37 (0.16–0.87)	0.02	
By sex									0.64
Male (n = 1,179)	129/600	5141.98	103/579	5090.72	0.81 (0.62–1.04)	0.10	0.89 (0.68–1.16)	0.39	
Female (n = 1,773)	69/878	8196.35	77/895	8342.29	1.10 (0.79–1.52)	0.58	1.02 (0.73–1.43)	0.90	
By baseline CVD history									0.76
No (n = 2,544)	118/1273	11,776.89	107/1271	11,892.03	0.90 (0.69–1.17)	0.42	0.94 (0.72–1.22)	0.63	
Yes (n = 408)	80/205	1561.44	73/203	1540.99	0.93 (0.68–1.28)	0.65	0.89 (0.63–1.26)	0.52	
By baseline diabetes medication use									0.80
No (n = 381)	17/196	1823.72	17/185	1746.97	1.03 (0.53–2.03)	0.92	1.69 (0.72–3.97)	0.23	
Yes (n = 2,544)	181/1269	11,388.93	162/1275	11,559.70	0.88 (0.71–1.09)	0.25	0.95 (0.77–1.18)	0.66	
By diabetes duration									0.40
≤ 10 years (n = 2356)	145/1195	10,896.10	128/1161	10,682.20	0.90 (0.71–1.14)	0.38	1.03 (0.80–1.31)	0.84	
> 10 years (n = 596)	53/283	2442.24	52/313	2750.82	0.87 (0.60–1.28)	0.49	0.71 (0.47–1.09)	0.12	
**Hp2-2 Phenotype**									
By race									0.97
White (n = 1197)	79/584	5313.27	92/613	5453.89	1.14 (0.84–1.54)	0.40	0.95 (0.69–1.31)	0.75	
Black (n = 167)	10/73	651.95	8/94	855.53	0.61 (0.24–1.54)	0.29	0.76 (0.17–3.38)	0.72	
Hispanic (n = 165)	10/81	727.21	6/84	794.97	0.55 (0.20–1.53)	0.25	0.60 (0.16–2.26)	0.45	
By sex									0.53
Male (n = 684)	64/319	2778.16	69/365	3135.29	0.95 (0.68–1.33)	0.77	0.86 (0.59–1.24)	0.41	
Female (n = 906)	42/448	4162.30	43/458	4238.55	1.01 (0.66–1.54)	0.97	1.04 (0.66–1.64)	0.87	
By baseline CVD history									0.15
No (n = 1377)	78/678	6237.99	61/699	6476.38	0.75 (0.54–1.05)	0.10	0.78 (0.55–1.10)	0.16	
Yes (n = 213)	28/89	702.48	51/124	915.47	1.39 (0.88–2.21)	0.16	1.18 (0.67–2.05)	0.57	
By baseline diabetes medication use									0.56
No (n = 219)	6/106	996.56	10/113	1041.41	1.60 (0.58–4.42)	0.36	1.86 (0.55–6.27)	0.32	
Yes (n = 1357)	99/651	5853.58	101/706	6317.47	0.95 (0.72–1.25)	0.70	0.87 (0.65–1.17)	0.35	
By diabetes duration									0.59
≤ 10 years (n = 1258)	76/615	5623.09	74/643	5847.15	0.94 (0.68–1.29)	0.69	0.84 (0.60–1.17)	0.30	
> 10 years (n = 332)	30/152	1317.37	38/180	1544.70	1.09 (0.67–1.75)	0.74	1.09 (0.61–1.94)	0.77	

*CAD* coronary artery disease, *CI* confidence interval, *CVD* cardiovascular disease, *DSE* diabetes support and education, *HDL-C* high-density lipoprotein cholesterol, *Hp* haptoglobin, *aHR* adjusted hazard ratio, *uHR* unadjusted hazard ratio, *ILI* intensive lifestyle intervention

^a^The CAD event outcome is a composite of fatal and non-fatal MI, hospitalization for angina, and possible fatal CAD

^b^Models were adjusted for age, sex, race, study site, prior history of CVD, triglycerides, systolic blood pressure, diastolic blood pressure, income, education, antidepressant medication use, any diabetes medication use, any anti-hypertensive medication use and any lipid medication use, except for when stratified by one of these variables. *P*-value threshold of < 0.002 (0.05 divided by 24) was used

^†^ P-value for the interaction between intervention and race, sex, history of CVD at baseline, diabetes medication use at baseline, or diabetes duration among each phenotype group for the adjusted model

In an additional sensitivity analysis, ILI was not associated with CVD, total mortality, or severe hypoglycemia when compared to DSE for either phenotype group (Additional file 1: Table S1). When restricted to years 1, 3 and 5, ILI was not associated with CAD risk for either phenotype group overall (Additional file 1 Table S2).

## Discussion

We previously found that intensive glycemic control (targeting HbA_1c_ < 6.0%) versus standard therapy (targeting HbA_1c_ of 7.0–7.9%) was effective at preventing incident CAD events among ACCORD study participants with the Hp2-2 phenotype while there was no association among participants without the Hp2-2 phenotype [16]. In the present analysis, we aimed to determine if the Look AHEAD study lifestyle intervention focusing on weight loss influenced risk of CAD events in a similarly Hp phenotype-dependent manner. We found that when compared to DSE, the ILI was not associated with risk of CAD among participants with or without the Hp2-2 phenotype. The ILI also did not result in substantial lowering of HbA_1c_ for either Hp phenotype group.

Differences between the ACCORD and Look AHEAD studies may help explain the current findings. The ACCORD study investigated the effect of intensive glycemic control (targeting HbA_1c_ < 6.0%) on CVD events compared to standard therapy (targeting HbA_1c_ of 7.0–7.9%) [14] while the goal of the Look AHEAD study was to test the effect of a lifestyle intervention for weight loss (caloric restriction and increased physical activity) on CVD events [4]. The ACCORD study was focused on the improvement of a single risk factor (blood glucose levels), while the Look AHEAD intervention targeted a broader range of risk factors (obesity, hypertension, blood glucose etc.). Although the ILI in the Look AHEAD study demonstrated improvement of a number of CVD risk factors when compared to DSE [4], the magnitude of improvement dwindled over time. In particular, the difference in mean HbA_1c_ levels between treatment arms was substantially less than the difference observed between treatment groups in the ACCORD study, and the lowest median HbA1c % of the Look AHEAD study treatment group (median of 7.2% at year 1 which was not maintained after year 1) was comparable to the control group of the ACCORD study (mean of 7.5% achieved in the control group at year 1 maintained for the study duration).

In the ACCORD study, HbA_1c_ between treatment groups differed substantially at year 1 (median of 6.4% in the intensive therapy arm versus 7.5% in the standard therapy arm) and this difference was maintained for the remainder of the study [14]. In the Look AHEAD study, the largest difference in mean HbA_1c_ of 0.6% (6.6% in the ILI arm and 7.2% in the DSE arm) between treatment groups was observed at year 1 and diminished over time with a difference of < 0.2% by midway through the trial [4]. Even the peak difference in HbA_1c_ between treatment arms in Look AHEAD did not reach the difference that was observed between treatment groups throughout the ACCORD study. When restricting our analysis to year 1 when the greatest difference in HbA_1c_ between treatment arms was observed in the Look AHEAD study, there was still no evidence to suggest that the effect of ILI was dependent on Hp phenotype (Additional file 1 Table S2).

Blood glucose levels at baseline were also lower in the Look AHEAD study participants compared to the ACCORD study participants (mean HbA_1c_ of 7.2% versus a mean of 8.3% respectively). As such, the Look AHEAD study participants in the ILI group may not have had sufficient glucose lowering compared to the DSE group to have influenced CAD risk in either Hp phenotype group. In accordance with this hypothesis, in the ACCORD study, we also found that the reduced risk associated with intensive therapy among participants with the Hp2-2 phenotype was likely attributed to participants not having high HbA_1c_ (≥ 8.0%) rather than achieving strict glycemic control and did not support a glycemic target of < 7.0% for either phenotype group [28].

The biological mechanism linking Hp phenotype and CAD is well supported in the scientific literature [7, 11–13, 29–31], and is specific to the setting of hyperglycemia. In brief, it is established that people with the Hp2-2 phenotype (compared to people without) produce a Hp protein that is larger and less effective at removing oxidative hemoglobin (Hb) from the blood (a primary function of Hp). This difference is magnified the more that Hb is glycated, with studies showing that HbA_1c_ ≥ 6.5% may be a key level of glycemia associated with oxidative Hb-Hp complexes that are dysfunctional as antioxidants in people with the Hp2-2 phenotype [7, 11, 29, 32, 33]. The Hp2:HbA_1c_ complex oxidizes serum lipoproteins, increasing susceptibility to atherosclerosis and ultimately CAD [11, 13, 33–35]. Therefore, interventions to manage glycemic control may be particularly important for CAD prevention among people with the Hp2-2 phenotype to help reduce Hp2:Hb mediated oxidative damage to blood vessels. The null results of the current study where the ILI did not substantially reduce blood glucose to levels indicative of strict glycemic control (i.e. < 6.5%) align with the hypothesis that it is the relationship between Hp type and glycemic control (lowered HbA_1c_) that affects risk of CAD.

Another possible explanation for the lack of a significant difference between treatment groups by Hp phenotype is that the study lacked sufficient power. However, the Look AHEAD study is the largest and longest clinical trial in people with diabetes to date to investigate this question and our results are hypothesis generating and can be used in a future meta-analysis to determine if these results are conclusive.

Hp phenotype distribution varies according to ethnicity/geography [10] and in the current study, we saw that the two phenotype groups had different race/ethnic distribution (Table 1). Further, Hp phenotype frequencies were not in HWE. For this reason, many studies (including our previous ACCORD study [16]) have historically reported findings among only the largest race-based group which is usually White participants; however, we will no longer exclude participants based on their race/ethnicity, so we have stratified by race/ethnicity. Before Bonferroni correction, ILI was not associated with risk of CAD among White participants in either phenotype group. It was associated with a higher risk of CAD among Black participants and a lower risk of CAD among Hispanics with the non-Hp2-2 phenotype (the largest of the two phenotype groups); however, these findings were not significant after correction for multiple comparisons, which suggests that they may have been due to chance. The majority of participants in this study were White and the sample size for the race-based groups in the present study were small and so the need to study the influence of Hp type on the relationship between lifestyle interventions and risk of CAD in a more representative population remains a priority.

In the current study, we observed that a significant difference in mean weight between study groups was maintained for the duration of the study for the non-Hp2-2 phenotype group only. In a recent study investigating the relationship between Hp phenotype and diet-induced weight loss, the Hp1-1 phenotype was associated with greater improvements in abdominal obesity, plasma insulin levels, and insulin resistance when compared to the Hp2-1 and Hp2-2 phenotypes in women with obesity; however, weight/BMI change was not different between phenotypes [36]. Similarly, in another study intermittent fasting was associated with a greater reduction in waist circumference among overweight/obese people with the Hp1-1 phenotype when compared to people with the Hp2-1 and Hp2-2 phenotypes [37]. Taken together with the results of the current study, these findings suggest that Hp phenotype may influence the outcome of weight-loss interventions. The mechanism linking Hp phenotype and weight is not clear but may be related to Hp antioxidant function as inflammation and obesity/weight gain are deeply intertwined and the Hp2-2 phenotype has less antioxidant capabilities compared to the non-Hp2-2 phenotypes [34]. Further investigation on the relationship between Hp phenotype and weight loss is warranted.

Our study had several limitations worth noting. The present cohort consisted of mostly White participants with type 2 diabetes who were motivated to lose weight through a lifestyle intervention and who could successfully complete a maximal-fitness test at baseline; therefore, the generalizability of our results to other populations is limited. In particular, we were underpowered to provide precise estimates of our primary association when stratifying by race/ethnicity and cannot rule out the possibility of chance findings within the race-based subgroups, which were no longer significant after correction for multiple comparisons. The educational sessions in the control group (focused on diet, exercise, and social support) may have lessened the difference in HbA_1c_ and outcomes observed between the two treatment groups in Look AHEAD compared to if there had been no educational sessions. Although our study allowed for adjustment for many potential confounders, other unmeasured confounders may be present. We were also underpowered to detect the association between the lifestyle intervention and stroke by Hp phenotype, and further research on this relationship is warranted. These analyses were not planned as part of the original Look AHEAD study protocol and thus should be considered exploratory.

In summary, we did not find any evidence to suggest the effect of an intensive lifestyle intervention for weight loss (focused on caloric restriction and increased physical activity) on CAD risk is dependent on Hp phenotype in the Look AHEAD study. The null results of the current study where blood glucose levels were not very different between treatment groups are consistent with the hypothesis that it is the relationship between Hp phenotype and glycemic control that affects risk of CAD; however, further research is needed to determine if these results are conclusive.

### Supplementary Information

**Additional file1: Table S1.** Multivariable adjusted hazard ratios (aHR) for other outcome events comparing assignment to intensive lifestyle intervention versus diabetes support and education control groups for each phenotype group separately. **Table S2.** Multivariable adjusted hazard ratios (aHR) for CAD events comparing assignment to intensive lifestyle intervention (ILI) versus diabetes support and education (DSE) for each phenotype group overall and in White participants only restricting follow-up to years 1, 3 and 5.

## Data Availability

The Look AHEAD datasets analyzed during the current study are available from the National Institute of Diabetes and Digestive and Kidney Diseases Central Repository, https://repository.niddk.nih.gov/studies/look-ahead/.The Hp phenotype dataset generated during the current study are available from the corresponding author on reasonable request.

## Abbreviations

ACCORD: Action to Control Cardiovascular Risk in Diabetes
BMI: Body mass index
CAD: Coronary artery disease
CI: Confidence interval
CVD: Cardiovascular disease
DSE: Diabetes support and education
ELISA: Enzyme linked immunosorbent assay
Hb: Hemoglobin
HbA1c: Glycated hemoglobin
HDL: High-density lipoprotein
Hp: Haptoglobin
HR: Hazard ratio
HWE: Hardy Weinberg equilibrium
ILI: Intensive lifestyle intervention
Look AHEAD: Action for Health in Diabetes
