# Supplementary material for: Intensive lifestyle intervention in type 2 diabetes and risk of incident coronary artery disease for the common haptoglobin phenotypes: the Look AHEAD study
Source: Cardiovasc Diabetol. 2024 Feb 24;23:82. doi: 10.1186/s12933-024-02164-8 (PMC10894470; doi:10.1186/s12933-024-02164-8)
Supplement: Supplementary file 1 — Additional file1: Table S1. Multivariable adjusted hazard ratios (aHR) for other outcome events comparing assignment to intensive lifestyle intervention versus diabetes support and education control groups for each phenotype group separately. Table S2. Multivariable adjusted hazard ratios (aHR) for CAD events comparing assignment to intensive lifestyle intervention (ILI) versus diabetes support and education (DSE) for each phenotype group overall and in White participants only restricting follow-up to years 1, 3 and 5. [file 12933_2024_2164_MOESM1_ESM.docx]

**Supplementary Table 1.** Multivariable adjusted hazard ratios (aHR) for other outcome events comparing assignment to intensive lifestyle intervention versus diabetes support and education control groups for each phenotype group separately.

|  | **DSE (Reference)** | |  | **ILI** | |  |  | **Hazard Ratios (HRs)** | |  |
| --- | --- | --- | --- | --- | --- | --- | --- | --- | --- | --- |
|  | **# Events/n** | **Person-years** |  | **# Events/n** | **Person-years** |  | **uHR (95% CI)** | **P-value** | **aHR(95% CI)** ^†^ | **P-value** |
| **Non-Hp2-2 Phenotype** |  |  |  |  |  |  |  |  |  |  |
| CVD* | 233/1478 | 13199.95 |  | 223/1474 | 13244.16 |  | 0.95 (0.79-1.15) | 0.61 | 1.02 (0.84-1.23) | 0.87 |
| Total mortality | 88/1478 | 14227.60 |  | 79/1474 | 14171.04 |  | 0.90 (0.67-1.22) | 0.51 | 0.95 (0.70-1.30) | 0.77 |
| Severe hypoglycemia** | 85/1478 | 13934.62 |  | 73/1474 | 13860.35 |  | 0.86 (0.63-1.18) | 0.36 | 0.81 (0.59-1.12) | 0.20 |
| **Hp2-2 Phenotype** |  |  |  |  |  |  |  |  |  |  |
| CVD* | 124/767 | 6845.50 |  | 132/823 | 7306.62 |  | 1.00 (0.78-1.28) | 0.99 | 0.91 (0.70-1.18) | 0.46 |
| Total mortality | 49/767 | 7349.74 |  | 46/823 | 7872.85 |  | 0.88 (0.59-1.31) | 0.53 | 0.74 (0.49-1.12) | 0.16 |
| Severe hypoglycemia** | 32/767 | 7241.74 |  | 36/823 | 7699.79 |  | 1.06 (0.66-1.71) | 0.81 | 1.07 (0.65-1.76) | 0.80 |

CVD=cardiovascular disease; CI= confidence interval; DSE= diabetes support and education; Hp= haptoglobin; aHR= adjusted hazard ratio; uHR= unadjusted hazard ratio; ILI= intensive lifestyle intervention

The P-values for the test of interaction between intervention and Hp phenotype for the adjusted model for each outcome are 0.45 (total CVD), 0.41 (total mortality), 0.44 (any hypoglycemia), 0.37 (severe hypoglycemia).

*****The CVD event outcome is a composite of death from cardiovascular causes, nonfatal mi, nonfatal stroke and hospitalization for angina.

** A Severe hypoglycemia event was defined as a loss of consciousness, seizure, or a glucose <70mg/dL that prevented self-treatment, and required assistance of another person

^†^Models were adjusted for age, sex, race, study site, prior history of CVD, triglycerides, systolic blood pressure, diastolic blood pressure, income (with category for missing), education, antidepressant medication use, any diabetes medication use, any anti-hypertensive medication use and any lipid medication use.

|  | **DSE (Reference)** | |  | **ILI** | |  |  | **Hazard Ratios (HRs)** | |  |
| --- | --- | --- | --- | --- | --- | --- | --- | --- | --- | --- |
|  | **# Events/n** | **Person-years** |  | **# Events/n** | **Person-years** |  | **uHR (95% CI)** | **P-value** | **aHR (95% CI)**** | **P-value** |
| **Non-Hp2-2 Phenotypes** |  |  |  |  |  |  |  |  |  |  |
| **Follow- up restricted to year 1** |  |  |  |  |  |  |  |  |  |  |
| Overall | 16/1478 | 1470.10 |  | 9/1474 | 1469.12 |  | 0.56 (0.25-1.27) | 0.17 | 0.59 (0.23-1.51) | 0.27 |
| White participants | 9/914 | 910.42 |  | 5/919 | 915.95 |  | 0.55 (0.19-1.65) | 0.29 | 0.40 (0.09-1.75) | 0.23 |
| **Follow- up restricted to years 1-3** |  |  |  |  |  |  |  |  |  |  |
| Overall | 60/1478 | 4350.22 |  | 39/1474 | 4363.49 |  | 0.65 (0.43-0.97) | 0.04 | 0.71 (0.46-1.08) | 0.11 |
| White participants | 42/914 | 2688.16 |  | 24/919 | 2720.56 |  | 0.56 (0.34-0.93) | 0.03 | 0.53 (0.32-0.90) | 0.02 |
| **Follow- up restricted to years 1-5** |  |  |  |  |  |  |  |  |  |  |
| Overall | 100/1478 | 7142.30 |  | 85/1474 | 7178.60 |  | 0.85 (0.63-1.13) | 0.26 | 0.87 (0.65-1.18) | 0.38 |
| White participants | 72/914 | 4397.18 |  | 56/919 | 4470.74 |  | 0.76 (0.54-1.08) | 0.13 | 0.71 (0.49-1.02) | 0.07 |
| **Hp2-2 Phenotype** |  |  |  |  |  |  |  |  |  |  |
| **Follow- up restricted to year 1** |  |  |  |  |  |  |  |  |  |  |
| Overall | 9/767 | 763.11 |  | 8/823 | 819.66 |  | 0.83 (0.32-2.14) | 0.70 | 0.87 (0.29-2.67) | 0.81 |
| White participants | 7/584 | 580.57 |  | 7/613 | 609.68 |  | 0.95 (0.33-2.72) | 0.93 | 0.73 (0.19-2.74) | 0.64 |
| **Follow- up restricted to years 1-3** |  |  |  |  |  |  |  |  |  |  |
| Overall | 25/767 | 2264.74 |  | 26/823 | 2430.97 |  | 0.97 (0.56-1.68) | 0.91 | 0.79 (0.43-1.45) | 0.44 |
| White participants | 17/584 | 1725.29 |  | 23/613 | 1806.11 |  | 1.29 (0.69-2.42) | 0.42 | 0.97 (0.47-1.99) | 0.93 |
| **Follow- up restricted to years 1-5** |  |  |  |  |  |  |  |  |  |  |
| Overall | 45/767 | 3717.65 |  | 54/823 | 3983.27 |  | 1.12 (0.75-1.66) | 0.57 | 0.94 (0.62-1.45) | 0.79 |
| White participants | 31/584 | 2839.28 |  | 47/613 | 2951.91 |  | 1.46 (0.93-2.30) | 0.10 | 1.23 (0.75-2.02) | 0.42 |

**Supplementary Table 2.** Multivariable adjusted hazard ratios (aHR) for CAD^*^ events comparing assignment to intensive lifestyle intervention (ILI) versus diabetes support and education (DSE) for each phenotype group overall and in White participants only restricting follow-up to years 1, 3 and 5.

CAD= coronary artery disease; CI= confidence interval; DSE= diabetes support and education; Hp= haptoglobin; aHR= adjusted hazard ratio; uHR= unadjusted hazard ratio; ILI= intensive lifestyle intervention

*The CAD event outcome is a composite of fatal and non-fatal MI, hospitalization for angina, and possible fatal CAD.

**Models were adjusted for age, sex (for overall only), race, study site, prior history of CVD, triglycerides, systolic blood pressure, diastolic blood pressure, income, education, antidepressant medication use, any diabetes medication use, any anti-hypertensive medication use and any lipid medication use.

P-values for the interaction between intervention and Hp phenotype for was 0.52 (overall) and 0.33 (White participants) at year 1, 0.44 (overall) and 0.06 (White participants) for years 1-3, and 0.53 (overall) and 0.06 (White participants) for years 1-5.
